# Significant Spontaneous Pneumomediastinum and Extensive Subcutaneous Emphysema in a COVID-19 Patient

**DOI:** 10.3390/reports7010015

**Published:** 2024-02-19

**Authors:** Arianna Gaspari, Francesca Carrieri, Matteo Villani, Elisabetta Bertellini

**Affiliations:** 1 Department of Anaesthesia and Intensive Care, Azienda Ospedaliero Universitaria di Modena and Reggio Emilia, Via Del Pozzo 71, 41211 Modena, Italy; arianna.gaspari@aou.mo.it (A.G.); frecarrieri@gmail.com (F.C.); 2 Department Anaesthesia and Intensive Care, Azienda USL Piacenza, Ospedale “Guglielmo da Saliceto” 49, 29121 Piacenza, Italy; mvillani@live.it

**Keywords:** COVID-19, spontaneous pneumomediastinum, Macklin effect

## Abstract

A 64-year-old man, who had no pre-existing health conditions, was admitted to the intensive care unit due to progressive shortness of breath resulting from COVID-19. Initially, the patient responded to non-invasive mechanical ventilation, which improved his breathing function. However, after six days, his respiratory function worsened significantly, requiring invasive ventilation. Out of nowhere, the person experienced spontaneous pneumomediastinum and extensive subcutaneous emphysema. The next day, a spontaneous pneumothorax occurred and was successfully drained later. It became evident that there was extensive subcutaneous emphysema also. The CT scan of the chest confirmed the presence of spontaneous pneumomediastinum, but it did not have any effect on the patient’s hemodynamics. The physicians performed a bronchoscopy and imaging with a contrast medium, which ruled out any lesions to the trachea or esophagus. No other issues related to the problem were identified during the examination. Unfortunately, microscopic bullae of interstitial emphysema, or micro air leaks, are visible when they are an adequate size on computed tomography. Recent literature and studies not available during the case report have shown that if the Macklin effect is detected on the baseline CT scan, it could predict the occurrence of pneumothorax or pneumomediastinum.

## 1. Background

COVID-19 patients are at an increased risk of developing spontaneous pneumomediastinum (SPM) and pneumothorax (PNX). These types of complications are life-threatening conditions and have a higher incidence of occurrence in patients with COVID-19 compared to the general population. It is defined by air in the mediastinum without any preceding trauma, surgical or medical procedure (including mechanical ventilation), hollow viscus perforation, or gas-producing infection [1,2]. The incidence of pneumomediastinum is extremely rare in patients undergoing noninvasive mechanical ventilation (NIV); however, patients with COVID-19 have a higher risk of developing spontaneous pneumomediastinum while on NIV [3]. It has been reported that SPM is a type of lung injury that can be caused by barotrauma. The rate of barotrauma in COVID-19 patients who require mechanical ventilation is 15%, which is higher than the rate observed in non-COVID-19 patients. Severe acute respiratory syndrome and acute respiratory distress syndrome (ARDS) can cause similar rates of 25%, and the rate in patients with ARDS and acute lung injury can range from 10% to 67% [4].

SPM can be triggered by vomiting or coughing; the latter is a common symptom in COVID-19 patients. The classic triad of pneumomediastinum includes dyspnea, retrosternal chest pain (typically pleuritic), and subcutaneous emphysema. Other symptoms may include neck pain, cough, dysphagia, and odynophagia. A chest CT is the most accurate way to diagnose pneumomediastinum.

## 2. Case Presentation

**Figure 1:**
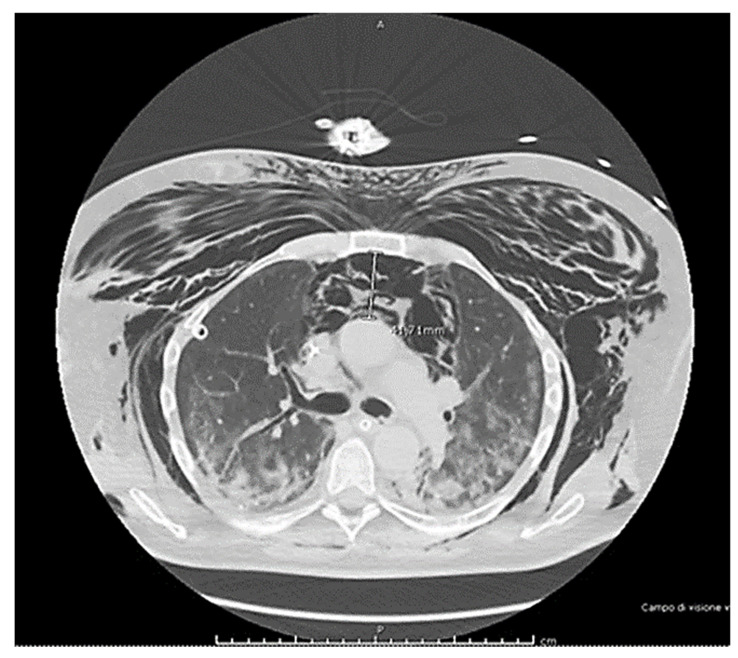
The imaging report shows extensive subcutaneous emphysema and a large spontaneous pneumomediastinum (>41 mm) in COVID-19 patients. Bronchoscopy exams found no evidence of lesions to the trachea or esophagus.

A COVID-19 patient, a 64-year-old man with no pre-existing health conditions, a non-smoker, no emphysema, or any chronic therapy, was admitted to the intensive care unit due to progressive shortness of breath. Initially, the patient responded to non-invasive mechanical ventilation, which helped improve his breathing function, but after six days, his respiratory function deteriorated significantly, requiring invasive ventilation. Suddenly, there was a development of SPM and extensive subcutaneous emphysema, and the next day, a spontaneous pneumothorax occurred, which was successfully drained. After that, it became evident that there was extensive subcutaneous emphysema. The chest CT scan confirmed the presence of spontaneous pneumomediastinum, but it did not affect the patient’s hemodynamics (Figure 1). The physicians performed a bronchoscopy and imaging with a contrast medium, which ruled out any lesions to the trachea or esophagus. No other issues related to the problem were identified during the examination. Unfortunately, microscopic bullae of interstitial emphysema, or micro air leaks, are visible when they are an adequate size on computed tomography [5]. Micro air leaks can be identified through CT scans, and one such effect that can be observed is the Macklin effect. Recent literature and studies not available during the case report demonstrated that if the Macklin effect is detected at the CT scan baseline, it could predict the occurrence of pneumothorax or pneumomediastinum [6,7]. The patient survived after six months of hospitalization and was discharged to a different facility for respiratory rehabilitation to treat long-term COVID-19 complications.

**Figure 2:**
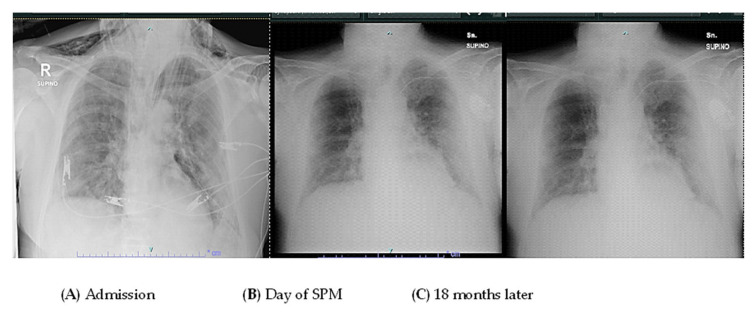
The figure reports a chest radiograph of the patient at admission (**A**) at the day of the SPM (**B**) and 18 months later (**C**).

**Figure 3:**
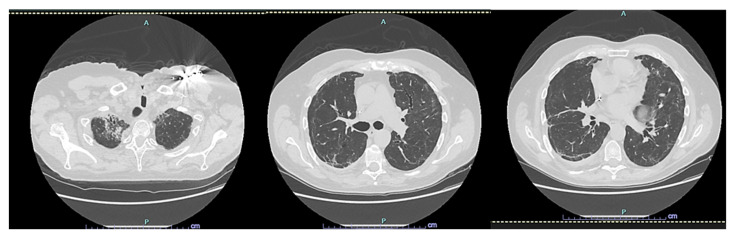
CT scan HRCT of the patient later, at 24 months.

After two years, the patient is still alive but has not fully regained respiratory function. However, their Barthel Index score was above 80% (Figure 2 and Figure 3).

## 3. Discussion

Individuals with COVID-19 who develop spontaneous pneumomediastinum (SPM) tend to have higher rates of mechanical ventilation, an increased likelihood of ICU admission, and longer hospital stays. Respiratory infections, such as COVID-19, can damage the alveolocapillary membrane, increasing the risk of alveoli rupture and leading to SPM [8,9]. Currently, there is no sufficient evidence to suggest discontinuing Continuous Positive Airway Pressure (CPAP) treatment when pneumomediastinum occurs in patients. The complication is often described as a care report. A larger, international dataset could be crucial in understanding and describing this problem. Further research is required in the field of SPM or barotraumatic events and the Macklin effect, and understanding the role of mechanical ventilation is crucial to prevent and avoid this complication. Recently, the CoBif score has been investigated as a predictor of mortality in clinical settings and to simplify the identification and management of high-risk patients [10,11,12,13]. Therefore, to prevent pressure gradients caused by positive pressure ventilation, one should focus on reducing airway pressures in cases of pneumomediastinum. There are few explanations for spontaneous barotraumatic events in COVID-19 patients, and experimental models are too complicated to replicate these types of complications. Investigations into similar barotraumatic events, such as those in acute respiratory distress syndrome (ARDS) caused by bacterial or viral infections, may provide additional insights [14,15]. Moreover, understanding the phenotype of ARDS could enhance the comprehension of the cause of micro-air leaks [16,17,18,19]. SPM does not always need to be treated, such as in the case of a benign, self-limiting condition, which could be conservatively treated [20]. Early decision making on the need and escalation for invasive mechanical ventilation is crucial to avoid complications such as patient self-inflicted lung injury and barotrauma-related sequelae like pneumothorax and pneumomediastinum [21]. NIV in certain COVID-19 pneumonia patients has no overall benefit in avoiding intubation, as confirmed by a recent systematic review [22]. While invasive mechanical ventilation may be associated with higher rates of barotrauma, this should not mean that intubation and invasive mechanical ventilation should be delayed [23].

When SPM occurs along with thorax compression and massive subcutaneous emphysema, it can lead to progressive hypoxemia and hypercapnia. In such cases, prompt drainage is a crucial maneuver. Various techniques exist for mediastinal decompression, including an incision with blunt finger dissection, needle aspiration, a percutaneous drainage catheter with continuous suction, and a mediastinal chest tube drain placement [24].

## 4. Strength and Limitations

The study provides valuable insights into the clinical manifestations and complications of COVID-19, particularly focusing on spontaneous pneumomediastinum and subcutaneous emphysema. Anyway, this case report and topic could be relevant given the ongoing impact of the pandemic, and could contribute to the understanding of COVID-19’s respiratory complications. Future research could benefit from larger sample sizes or a review of multiple cases to comprehensively understand the prevalence and risk factors.

## 5. Conclusions

If you suspect that someone has COVID-19 and they also experience chest pain or difficulty breathing, it is important to consider spontaneous pneumomediastinum (SPM) as a possible diagnosis. Early detection of SPM through a CT scan could be recommended, as SPM can be a serious complication in COVID-19 patients. It is particularly important to check for the possible occurrence of the Macklin effect in these patients. An interesting perspective could be provided by the CoBif score, and in the future, a machine learning approach could be used to evaluate these factors as a possible predictive system for preventing or calculating the risk of SPM.

## Data Availability

Not applicable.
